# Sussex Lunatic Asylum

**Published:** 1859-07-01

**Authors:** 


### SUSSEX LUNATIC ASYLUM.

### HOW THE INMATES WILL BE LODGED, FED, CLOTHED, EMPLOYED, AND AMUSED.

During the two years that this structure has been in course of erection, we have taken occasion to give Brighton people—and it must be remembered that our town alone contributes one-fifth to the cost of it—an idea both of its character and extent. Our readers know that it stands on a farm of 120 acres, in the parish of Wivelsfield, at the southern extremity of Hayward's Heath, and about a mile from the station. They know it is a building in what we suppose must be called the Italian style, to which a degree of novelty is imparted by the use of parti-coloured bricks, conveying the idea of simple-chromatic decoration. They are aware, also, that the frontage is 850 feet in extent; that the structure consists of a centre-building and two wings; and that there is, in addition, a detached church, which rumour speaks of, and without exaggeration, as the prettiest in the county. This and much more having already been explained, we do not at present intend to detain the reader with that kind of information which is derived from plans and sections and architectural "views;" but rather to convey an idea or two of the accommodation which the inmates of this asylum are to receive—how, in fact, they will be housed, fed, clothed, taught, amused, and, in many cases, let us hope, cured.

Lunatic accommodation in the olden times was a simple matter enough. Such and such strength of dungeon, so much length of chain, such and such allowance of straw, of bread and of water; and there was an end of it. If the poor wretch raged, they tortured him; if he pined, he might, if it pleased him, pine to death. The asylum was like that place of horror over the door of which the Italian poet wrote that those who entered were to abandon even Hope. How all this has changed in our day, we need hardly say. The experiment first tried at Hanwell, of discipline without restraint, has produced results alike cheering and overwhelming. Half the horror of the most horrible of human calamities has passed away since it no longer exposes its victim to the brutality of ignorance, but renders him an object of generous and enlightened solicitude. And it is to this change in the aspect of the question that we owe our Sussex Asylum, and all the arrangements and provisions we are about to describe.

On visiting this building the other day, our first desire was to see the ordinary living and sleeping rooms of the inmates. This will probably be the first object of curiosity with our readers also. Supposing that to be the case, and that they were being shown over by Mr. Mortlock, the intelligent superintendent, he would probably say to them as to us—"You will form a rough idea of the building by remembering that the central portion is chiefly devoted to the various offices connected with the institution; the whole of the west wing is devoted to the females; and the whole of the east wing is devoted to the males. We will proceed, first, to the west wing."

With this we enter a suite of rooms, one room opening into another, and the suite extending the entire length of that wing on the ground-floor. These are the day-rooms for the patients. Here they will sit and read, or sew, and will take their meals—for no dining-hall has been provided, it being found better that the patients should dine in their own wards—not in solitude, but in families of greater or less magnitude, according to the stage, condition, or peculiarities of the malady of each. These rooms are all roomy and cheerful; and all command a wide country view, not through gratings or loopholes, but from glazed windows, constructed so that the safety of the inmate is secured without the idea of confinement being conveyed. The rooms are simply but comfortably furnished, each article of furniture being grained in imitation of oak. What took us more by surprise than any other circumstance was, that these rooms, and, indeed, those throughout the building, are heated, not by any arrangement of steam or hot air, but by means of open grates similar to those in ordinary drawing-rooms. "But," we exclaimed, seeing these open grates, and chests of coals beside them ready for use, "can you trust the patients with fire? Is it safe?" "It is considered perfectly safe," was the reply; "and is both more healthful and more cheerful than any system of heating by means of pipes."

Out of these wards or day-rooms, open bed-rooms, to be used by the patients. These are of two kinds: there are single rooms for one inmate each, and dormitories which will accommodate six or eight. Both kinds are light and airy; the fittings are similar; but as the single rooms are intended for the more violent or dangerous patients, they are not quite so amply furnished. In a single room there is a birch-wood bedstead with sacking-bottom, hair mattress, and the usual bedding, &c. The floor is bare; but beside the bed lies a strip of carpet. The windows of these rooms can be secured by shutters on the inside. The dormitories, or larger rooms, have no shutters, and are in every respect like ordinary bed-rooms. The inmates of these rooms are furnished with washstands for their use, though there is a lavatory attached to each ward for general purposes, fitted in the most complete manner. It has rows of basins, with hot and cold water taps to each; a bath, either hot or cold; a shower-bath—in fact, every means has been adopted to secure the perfect cleanliness of the inmates. We should have mentioned that between each sleeping-room is a nurse's room, with a window on either side, overlooking the patients; obviously a very necessary precaution. In answer to a question, we were informed that there are two or three attendants on these wards; but that the bulk of the work necessarily arising out of the daily life of so many persons will be chiefly performed by the inmates. Under proper guidance, they are quite capable of light work, and it is better that they should be employed than remain in listless idleness.

The wards we have inspected will be occupied by those properly designated "patients;" but a large number will come under the category of "convalescents," and though unable to be sent abroad, or, at least, likely to benefit by remaining in the Asylum, they are quite capable of following the occupations to which they have been accustomed, or others which they may be taught. For the use of this class, the upper story of this wing is devoted to what is called a sleeping-gallery, but which is, in fact, a corridor, flanked with bed rooms like those on the first floor. These rooms, for the most part, contain eight beds. Among the most necessary occupations in the building will be those connected with the laundry, and in these the convalescent among the female inmates will be largely engaged. And the laundry arrangements (at the extremity of the wing) are very perfect. There are two large apartments filled with washing apparatus—with troughs, into which water, hot or cold, is admitted by taps, with boiling coppers, &c. In one of the wash-houses a "wringing-machine" has also been provided—a most ingenious contrivance, by which some of the hardest work in connection with the washing is saved. The linen is put wet into a cylindrical sieve, the sides of which are formed of wire, and by means of a handle and wheel this cylinder is made to revolve so rapidly that all the moisture from the linen is, in fact, whirled off. When wrung, the clothes are taken into a spacious drying-yard, fitted with copper-wires in place of clothes'-lines. There are also three hot closets, heated by means of pipes, for drying and airing clothes; a sorting-closet; in fact, every necessary for carrying on this branch of domestic economy in the most expeditious and satisfactory manner.

Thus much for the western wing, devoted to the female inmates. The east wing, for the men, is similar in its fittings in all respects, until you reach the extremity, and there, in place of the laundry, you find provision made for pursuits adapted to the male convalescent patients. There is the tailors' shop,

(with its indispensable board), the shoemakers' shop, the upholsterers' shop, the carpenters' shop, a bakehouse with its oven, a brewhouse, stables, and so forth. Thus many, if not all, the necessities of the Asylum will be supplied from lunatic labour. This also will extend beyond the interior. Already there are eleven acres of land under cultivation as a kitchen-garden, and in time the rest of the 120 acres on which the Asylum stands will constitute a farm in which every branch of labour will be performed by the insane, of course under proper training and *surveillance*. It will be a curious, yet a gratifying sight—all these farming operations progressing quietly and in perfect order, in the hands of those who but a few years since were deemed incapable of anything but violence and destruction!

In the little world of the Asylum, however—and a strange, fantastical little world enough it will be—labour must have its relief. There must be repose. There must be amusement. There must be devotion. And for each of these exigencies provision has been made.

For repose, as well as exercise, pleasant-terraced grounds have been provided for the use of the inmates. These spread out before the southern front of the building, and are ingeniously formed, so that, while really surrounded by a high wall, they do not appear to be enclosed. The grass plat rises in terraces, one above the other, and each terrace is raised above the wall, and commands a prospect, taking in the whole range of the South Downs from Lewes to the Dyke, a pleasant wooded country, dotted here and there with well-known villages.

These airing-grounds, as they are called,—and we may observe that there is one for each sex—will conduce both to repose of mind and invigoration of body. But it is found that the insane require something more. They need society and amusement; and with this view a Recreation Hall has been erected in the centre of the building, which will be used for lectures, music, dancing, and such modes of entertainment as are adopted in all the modern Asylums. The Hall is spacious, capable of holding some 300 persons, and presents a cheerful and pleasing appearance; the only drawback being that the roof, which is supported by pillars, is too low. In this Hall both sexes will mingle in the dance, or listening to music, or witnessing entertainments, and perhaps on the occasions of these *réunions* the poor unfortunates will be seen under the most favourable circumstances. Accounts have so often been given of the *soirées* at St. Luke's, Hanwell, and other large Asylums, that this is a phase of lunatic life with which the public are now quite familiar.

Less has been written upon the lunatic at church; but that also is a strange as well as a solemn spectacle. In this Asylum unusual attention has been paid to this matter, and the consequence is, that the Asylum Church is, as we have said, the most beautiful for its size of any in the county. And next to its beauty, one is struck by the fact of the simple means by which it has been obtained. The difference between a beautiful and an ugly structure is often put upon the ground of difference of cost. There is no greater fallacy. It is to taste rather than to money that we owe everything in architecture as in the rest of the arts. Now, the taste evinced in this matter by the architect (Mr. H. E. Kendall, of Brighton, we believe), is exquisite, and the result is, that we have a gem without extravagant outlay. The material of the church is brick; but it is of various colours, and those colours are artistically worked up. The architecture is simple; but the proportions are such as to please the eye. So, again, in regard to ornamentation and fittings—all is simple, but in keeping. As points that arrested our attention, we may note the open roof, stained in imitation of ancient oak and satin wood; the chancel, with the transparent roof of purple glass, through which stars appear to be shining. The painted windows, inexpensive, but serving to give a subdued "religious light," in which the homely-sculptured stone is as effective as marble would be under other circumstances. The chief specimen of sculpture, by the way, is the altar-piece, representing

Christ among the Disciples, with the legend, "And He was known to them by the breaking of bread." Other points might be specified; but we must only add that this church will accommodate about 300, and that the seats are open, in a manner precisely similar to those of most modern Gothic churches. One side of the sacred edifice will be devoted to the males, the other to the females, but they are not even separated by a central aisle.

Those curious as to the economy of the Asylum may ask—"How do the inmates dress?" Well, there is no uniform dress, as, in fact, the different classes of patients require different clothing; but the prevailing colours for garments are blue and grey, and the style is that ordinarily adopted by persons in the humbler walks of life.

"And as to culinary utensils, and so forth?"

They are of the customary description. We saw a large store of crockery, all of it white, with the Asylum stamp on it, and comprising plates, dishes, jugs, &c. Among other articles which arrested our attention were some dozens of earthenware spittoons. "You permit smoking in the Asylum?" we remarked. "Yes," was the reply, "many of the inmates of these establishments find great relief in the pipe." "You do not regard it as one of the predisposing causes of that state of mind which sends them here?" "No; or after what I have seen of madness I would never touch a pipe again." From the extensive beer-cellar disclosed to us, we gathered that the poor inmate is not denied the simple luxury of a glass of beer either.

We have almost exceeded the due length of our article; and have now left unnoticed various points, such as the lighting of the building from gas manufactured on the grounds; the water supply, by means of a well, water-towers, &c.; the extensive kitchen and storage arrangements necessary for the supply of the daily wants of 450 persons; the medical department, and so forth. We should also allude to the Infirmaries. These are two in number, one at either extremity of the building, and are furnished with every care for the comfort of those sick in body as well as in mind. While inspecting the male infirmary, we ventured to ask, upon an idea which had suddenly struck us—"In all that we have seen there is no provision for restraint: now, however perfect your system, you *must* sometimes have violent patients whose paroxysms it is impossible to restrain. How do you act?" Our guide answered the question fairly and promptly, by leading us into a room similar to the ordinary bedrooms. "This," said he, "will be padded: the walls, to a height of eight feet, and the floor will be covered with a sort of mattress padding, against which the patient may even dash his head without sustaining any injury." There will be no furniture here. A second room of a like kind was shown us, and we also saw two rooms in which there were wooden "cribs" instead of bedsteads, designed for epileptic patients, who are apt to be seized with paroxysms in the night.

With an acknowledgment of the courtesy with which we were received and shown over the Asylum, we must conclude this article; first, however, adding, for the public information, the names of the heads of the staff already appointed for the management of the Asylum. They are as follows:—

Medical Superintendent—Dr. Robertson. Steward and Clerk—Mr. Mortlock. Assistant-Surgeon—Mr. Guynne. Housekeeper—Mrs. Stroude.—(*Brighton Herald, June* 4.)

---

